# Robust coherent control of solid-state spin qubits using anti-Stokes excitation

**DOI:** 10.1038/s41467-021-23471-8

**Published:** 2021-05-28

**Authors:** Jun-Feng Wang, Fei-Fei Yan, Qiang Li, Zheng-Hao Liu, Jin-Ming Cui, Zhao-Di Liu, Adam Gali, Jin-Shi Xu, Chuan-Feng Li, Guang-Can Guo

**Affiliations:** 1 CAS Key Laboratory of Quantum Information, University of Science and Technology of China, Hefei, Anhui People’s Republic of China; 2 CAS Center for Excellence in Quantum Information and Quantum Physics, University of Science and Technology of China, Hefei, Anhui People’s Republic of China; 3 Department of Atomic Physics, Budapest University of Technology and Economics, Budapest, Hungary; 4 Wigner Research Centre for Physics, Budapest, Hungary

**Keywords:** Quantum information, Qubits

## Abstract

Optically addressable solid-state color center spin qubits have become important platforms for quantum information processing, quantum networks and quantum sensing. The readout of color center spin states with optically detected magnetic resonance (ODMR) technology is traditionally based on Stokes excitation, where the energy of the exciting laser is higher than that of the emission photons. Here, we investigate an unconventional approach using anti-Stokes excitation to detect the ODMR signal of silicon vacancy defect spin in silicon carbide, where the exciting laser has lower energy than the emitted photons. Laser power, microwave power and temperature dependence of the anti-Stokes excited ODMR are systematically studied, in which the behavior of ODMR contrast and linewidth is shown to be similar to that of Stokes excitation. However, the ODMR contrast is several times that of the Stokes excitation. Coherent control of silicon vacancy spin under anti-Stokes excitation is then realized at room temperature. The spin coherence properties are the same as those of Stokes excitation, but with a signal contrast that is around three times greater. To illustrate the enhanced spin readout contrast under anti-Stokes excitation, we also provide a theoretical model. The experiments demonstrate that the current anti-Stokes excitation ODMR approach has promising applications in quantum information processing and quantum sensing.

## Abstract (web summary)

Optically detected magnetic resonance of defect spins typically relies on Stokes excitation, in which the excitation energy is larger than that of the emitted photon. Here, the authors use the opposite regime of anti-Stokes excitation to detect Si vacancy spins in SiC, with a threefold improvement in signal contrast.

## Introduction

Color centers in wide-bandgap semiconductors have been widely used in quantum networks^1–5^, quantum information processing^6–10^, and quantum sensing^11–17^ owing to their stable fluorescence properties and long spin coherence time even at room temperature^10,13^. Spins of color centers can be polarized by laser and manipulated by microwave (MW), allowing a readout approach operating on spin-dependent fluorescence detection^1–10^. Optically detected magnetic resonance (ODMR) technology can be used on this basis to detect the spin state, which is the foundation for applications of quantum technology^1–17^. However, previously, almost all ODMR readout protocols have used conventional Stokes excitation, in which the wavelength of the excitation laser is shorter than the zero phonon lines (ZPLs) of the color centers. Investigation of additional spin readout protocols could significantly extend the scope of solid-state quantum technologies. For example, photocurrent detection of magnetic resonance (PDMR) has been demonstrated and implemented in both nitrogen-vacancy (NV) centers in diamond and defects in silicon carbide (SiC) based on spin-dependent ionization dynamics, which benefits device integration^18,19^. However, PDMR usually requires complex doping and nano-fabrication technologies, and the output signal has to be increased^18,19^.

On the other hand, the anti-Stokes (AS) excitation process, in which the wavelength of the exciting laser is longer than that of the emitted photons, has been observed in quantum dots, dye molecules, and rare-earth doped crystals suited for applications, including Raman spectroscopy and laser cooling^20,21^. The mechanisms of AS excitation have been clarified, such as multiphoton absorption, phonon absorption, and Auger recombination^20,21^. Most recently, it has been also observed in the color centers of diamond and defects in hexagonal boron nitride (hBN)^20,21^. These were shown to be helpful in all-optical temperature sensing and manipulating emissions of quantum emitters^20,21^. However, there are still no published studies on ODMR detection and coherent manipulation of solid-state spin states under AS excitation. The realization of spin readout with AS excitation would broaden the boundary of quantum technologies based on solid-state spin qubits.

In recent years, color centers in SiC have been studied as promising candidates for bright single photon sources^22,23^ and spin qubits^8–10^. In contrast to diamond, SiC is a technology-friendly semiconductor with mature micro–nano fabrication and inch-scale growth technologies. Optically addressable spin qubits, including silicon vacancy (V_Si_) defects^4,10^, divacancy^8,9^, NV centers^24–26^, in SiC have drawn much attention due to their near-infrared and even telecom-ranged fluorescence and long spin coherence time^4,9,10,24–26^. These superior characteristics make them extensively applicable to quantum information processing^8–10^, spin–photon interfaces^4,5^, and quantum metrology, such as magnetic field^15^, electric field^16^, and temperature^17^.

In this work, we realize the coherent control of V_Si_ defect ensemble spins in 4H-SiC under AS excitation at room temperature. First, AS photoluminescence (PL) is investigated as a function of AS exciting laser power, temperature, and duration times, showing that AS PL is caused by the phonon-assisted single photon absorption process. On this basis, the Stokes and AS excited ODMR spectra are obtained for different excitation laser power, MW power, and temperature, which are shown to follow similar behavior. We then measured the external magnetic field-dependent Stokes and AS ODMR, both of which exhibit the same Zeeman splitting phenomena. Finally, a comparison of the free induction decay and spin echo of V_Si_ defects under Stokes and AS excitation reveals that AS excitation does not affect the coherent properties of V_Si_ defects. Hence, the demonstrated AS excited ODMR approach shows potential for quantum information processing and quantum sensing.

## Results

V_Si_ defects are point defects containing a silicon vacancy, which exist in 4H-SiC and 6H-SiC. In 4H-SiC, two forms of V_Si_ defects exist due to discrepancies in the silicon vacancy lattice: V1 and V2 centers have corresponding ZPLs of 861 and 915 nm, respectively. Since the spin of the V2 center can be manipulated at room temperature, we focus only on the V2 center of the V_Si_ defect, which features a *S* = 3/2 spin quartet and 70 MHz ground state zero field splitting (ZFS). A home-made confocal system combined with a MW system is implemented to excite and manipulate the V_Si_ defect (see details in “Methods”). Lasers with wavelengths of 720 and 1030 nm are used for the Stokes and AS excitation of V_Si_ defects, respectively. A band-pass filter with a transmitting wavelength of 850–1000 nm is used in the confocal set-up. In the experiment, we use a 4H-SiC sample with high-density V_Si_ defect ensembles^27^ (see details in “Methods”).

**Fig. 1:**
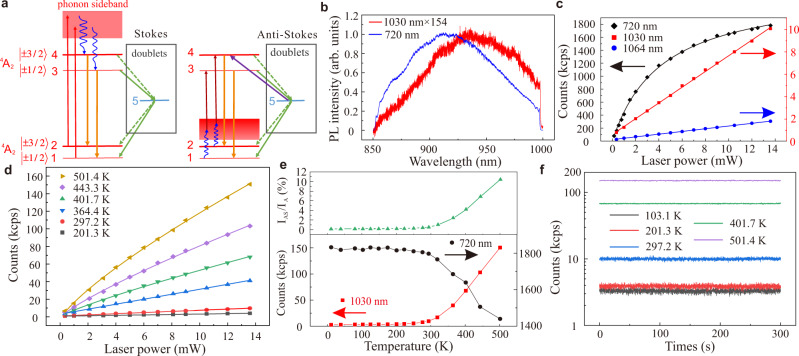
The AS excitation model and the AS PL of V_Si_ defects in 4H-SiC. **a** Excitation schemes of V_Si_ defects under Stokes excitation (left part) and anti-Stokes excitation (right part). The red (dark brown) arrows represent optical excitation from the ground state (labels 1 and 2) toward the excited states (labels 3 and 4) and the yellow arrows represent the associated spin-preserved radiative decay. The green arrows represent intersystem crossing (ISC) routes where dotted arrows mean weaker transitions than those represented by straight arrows. Blue wavy arrows show the phonon-related decay and excitation routes that are temperature dependent. The purple arrow represents a spin-flipping effective optical excitation from a metastable doublet state toward, selectively, to state 4. This is the key process in increasing the ODMR contrast upon anti-Stokes excitation, which is discussed in detail in the part of theoretical analysis. The energy differences are not linearly scaling for the sake of clarity. **b** The PL spectra of V_Si_ defects under Stokes (blue) and AS (red) excitation at room temperature, respectively. The units for both cases are equal, and the Stokes excited PL spectrum intensity is approximately 154 times larger than that of anti-Stokes. **c** The counts of V_Si_ defects as a function of laser power under Stokes (720 nm, black) and two different AS (1030 nm, red; 1064 nm, blue) excitations at room temperature, respectively. The corresponding lines represent the fits. **d** The counts of V_Si_ defects with respect to the laser power under AS excitation (1030 nm) at various temperatures. **e** The bottom panel displays the maximal counts with 13.6 mW Stokes (black) and AS (red) excitations as a function of temperature, respectively. The top panel shows the corresponding temperature-dependent ratio of AS to Stokes counts. **f** The AS PL time trace at five different temperatures with a time bin of 100 ms.

The proposed energy model for PL and optically induced spin polarization of the Stokes and AS excitation of V_Si_ defect in 4H-SiC is shown in Fig. 1a^20,21,28–31^. The defect has *S* = 3/2 ground and optically accessible excited state with *m*_s_ = ±1/2 and *m*_s_ = ±3/2 spin manifolds. The ground state and the lowest energy *S* = 3/2 excited state have ^4^A_2_ symmetry. In this case, we use a five-level scheme where the series of doublet states is substituted by a single effective doublet state (state 5). The electron may scatter from the excited state spin manifolds (states 3 and 4, i.e., *m*_s_ = ±1/2 and *m*_s_ = ±3/2, respectively) to this state or can scatter from this state to the ground state spin manifolds (states 1 and 2, i.e., *m*_s_ = ±1/2 and *m*_s_ = ±3/2, respectively). For the usual Stokes excitation (Fig. 1a left part), both the 1 and 2 ground states transmit to the excited states phonon sideband under the laser excitation. Generally, the simple phonon relaxation and excitation processes should be spin-conserving (only contain spin-independent operators). By phonon relaxation, they then rapidly thermalize to the 3 and 4 states respectively, followed by the spin-preserving radiative transition to the ground states (see Supplementary Note 1 for more details). In the AS excitation (Fig. 1a right part), the probability of going from the ground state manifold to the excited state manifold is less than that in the Stokes excitation, since it involves the phonon excitation in the former process. For both the Stokes and AS excitations, we assume that the non-radiative intersystem crossover to the metastable doublet state occurs primarily from state 3, then they polarized to state 1^30^.

We then investigated the AS PL of V_Si_ defects in 4H-SiC. The PL spectra of the V_Si_ defects under Stokes (720 nm, blue) and AS excitation is seen in Fig. 1b (1030 nm, red). Compared with the Stokes PL, the AS excited PL has a small red shift, which is consistent with previous results of NV centers in diamond^20^. To recognize the origin of the AS PL, we measure counts as a function of laser power (*P*) of the Stokes and two AS (1030 nm and 1064 nm) excitations (Fig. 1c). The Stokes PL is fitted using the function *I*(*P*) = *I*_s_/(1 + *P*_0_/*P*), where the maximal count *I*_s_ is 2350 kcps and the saturation power *P*_0_ is 4.3 mW, respectively. However, both of the AS excited counts follow a linear fit, and the counts with 1030 nm laser excitation are 5.5 times larger than that of the 1064 nm laser excitation. The linear laser power-dependent behavior indicates that lower energy single photons rather than double photons are absorbed in the AS excitation process. In comparison, the efficiency of AS excitation becomes higher when the excitation wavelength is closer to the ZPL, which is consistent with that of color centers in diamond^20,28^ and defects in hBN^21^. The temperature-dependent AS excitation is investigated to further validate that the AS excitation involves a phonon-assisted process. The AS count scales linearly with the exciting laser power for one-photon absorption, while it scales quadratically for two-photon absorption^20,21,28^. Figure 1d presents the AS excited counts with respect to laser power at different temperatures, where the counts increase quickly as the temperature rises. At temperatures <365 K, the counts linearly increase with laser power, which indicates that the AS PL is due to the phonon-assisted single low-energy photon absorption process (see Supplementary Note 2). In comparison, at temperatures >365 K, the data were fitted using a function of *bP*^*n*^ where the power index *n* decreases from 0.85 to 0.78 as the temperature increases from 401.7 to 501.4 K and *b* represents the temperature-dependent increasing parameter^21,28^. The decrease of the power index *n* might be attributed to the reduction in efficiency of the phonon-assisted single photon absorption process.

To quantify the AS excitation efficiency, we use the ratio *I*_AS_/*I*_S_ of AS (*I*_AS_) to Stokes (*I*_S_) excited counts^20^. A summary of the Stokes and AS excited counts as a function of the temperature is plotted in the lower panel in Fig. 1e. The Stokes excited count *I*_*S*_ is almost constant for temperatures varying from 11 to 300 K but declines rapidly as the temperature rises to 500 K. In comparison, when the temperature rises from 11 to 300 K, the AS excitation counts *I*_AS_ increase slowly and then increase rapidly as the temperature rises to 500 K. The ratio *I*_AS_/*I*_S_ increases slowly as the temperature increase to 300 K, which is shown in the upper panel in Fig. 1e. At room temperature, *I*_AS_/*I*_S_ is around 0.57%, which is approximately 50 times the ratio for NV centers in diamond^20^. *I*_AS_/*I*_S_ can be further increased using a shorter wavelength of AS excited laser^20,21^. In addition, *I*_AS_/*I*_S_ increases to 10.4% at 500 K, which is comparable with the silicon vacancy and germanium vacancy in diamond at room temperature^20^. The dramatic increase in the behavior of *I*_AS_/*I*_S_ at elevated temperatures will be useful for all-optical high temperature sensing^20^. Combined with SiC nano-particles^32^, nanoscale temperature sensors usable in living cells and microelectronic systems can be constructed. Through manufacturing photonics structures such as solid immersion lenses^10^ and so on, it is possible to realize AS excitation of a single V_Si_ defect^20^. We further investigated the stability of PL under AS excitation at various temperatures. Figure 1f displays the results with five representative temperatures, with all of the counts stable for these temperatures. We also note that the low PL photon counts for the AS approach would limit the application by employing only single emitters, while the quantum-sensing sensitivity can be dramatically increased by a factor of $\sqrt{N}$, where *N* is the number of the color centers^33^. Ensemble color centers would be useful for high-sensitivity quantum sensing.

**Fig. 2:**
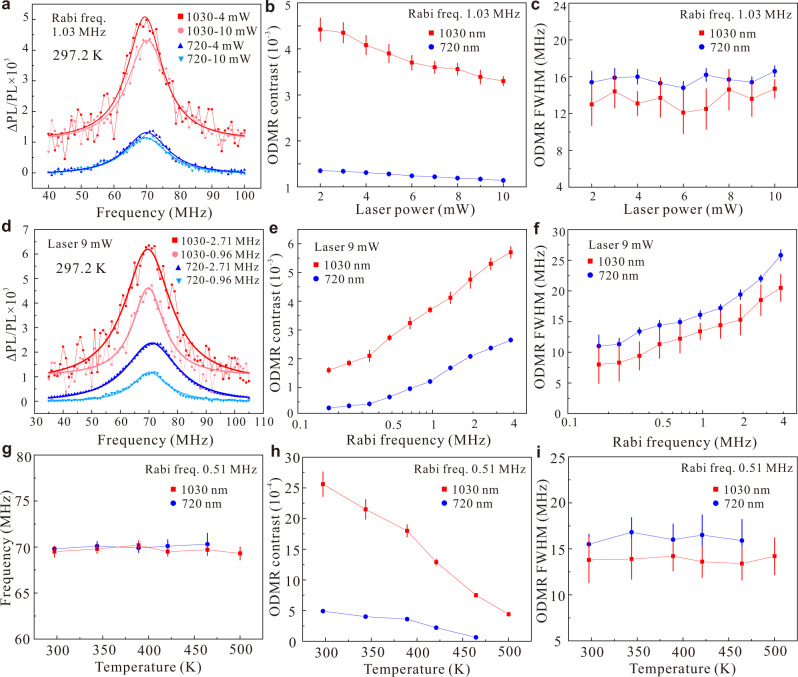
The ODMR spectra of V_Si_ defects under Stokes and AS excitation at zero external magnetic field. **a** Two representative Stokes and AS excited ODMR signals with different laser powers at room temperature (297.2 K). The lines are fitted using Lorentzian functions. **b**, **c** Comparison of Stokes and AS excited ODMR contrast and full width half maximum (FWHM) as a function of laser power, respectively. **d** Two representative Stokes and AS excited ODMR signals with different MW powers (Rabi frequencies) at room temperature (297.2 K). **e**, **f** Measurement of Stokes and AS excited ODMR contrast and FWHM as a function of Rabi frequency, respectively. **g** ZFS of V_Si_ defects under Stokes and AS excitation as a function of temperature. **h**, **i**, Comparison of Stokes and AS excited ODMR contrast and FWHM with respect to the temperature. Error bars represent the standard deviations of the corresponding fittings.

On the basis of stable phonon-assisted single photon emission-induced AS PL, we investigate AS excited ODMR of V_Si_ defect ensemble and compare it with Stokes excited ODMR. Figure 2a shows two representative Stokes and AS excited ODMR spectra at different laser powers with the same MW power, which is characterized by the Rabi frequency and the value is read as 1.03 MHz. The contrast of both the Stokes and AS excited ODMR at 4 mW laser power is slightly larger than at 10 mW. In addition, under AS excitation, the contrast is around three times that of Stokes excitation. Comparisons of ODMR contrast and linewidth as a function of laser power under Stokes and AS excitation are presented in Fig. 2b, c, respectively. Both the ODMR contrast decreases as the laser power increases, and the AS ODMR contrast remains around three times larger than the Stokes one. Meanwhile, both ODMR linewidths stay almost the same as laser power increases, in line with previous results^19^. The mean AS excited ODMR linewidth is around 2.2 ± 0.7 MHz smaller than that of the Stokes excitation. For two different MW powers, representative Stokes and AS excited ODMR signals at the same laser power are shown in Fig. 2d. Both the ODMR contrast and linewidth at higher Rabi frequency are larger than that at lower frequency, and the AS excited ODMR contrast values are also much higher than the Stokes ones. However, the ODMR signal-to-noise ratio (SNR) of the AS excitation is still low, which is due to the low AS excited counts and intrinsic low ODMR contrast of the silicon vacancy^10,27^. Unlike the Stokes excited counts that have smaller saturation power of the Stokes laser, the AS excited counts linearly increase as the AS exciting laser power. In the experiments, we can use a higher laser power (>400 mW) to significantly increase AS excited counts^34^. In addition, the thermally quenched methods will increase the intrinsic ODMR contrast by about ten times^34^. So, using the thermally quenched sample and higher AS exciting laser power, the ODMR SNR of the AS excitation will obviously increase^34^.

Figure 2e, f display a comparison of the ODMR contrast and linewidth under Stokes and AS excitation as a function of MW power. Both the ODMR contrast and linewidth increase as the MW power increases with similar behavior. In comparison, the AS excited ODMR contrast is much higher than that for Stokes excitation, while the linewidth is again lower than the Stokes excitation one. To understand this phenomenon, we assume that the AS excitation energy may excite the electron from a metastable doublet state, selectively, to state 4, which represents a spin-flipping effective optical excitation (see Fig. 1b). In this scenario, the brighter spin state is occupied in the quartet excited state manifold, which contributes to an enhanced AS excited ODMR contrast. We discuss the possible mechanism in detail in the theoretical part.

Since the AS excited PL counts increase rapidly as temperature increases, the temperature dependence of the AS excited ODMR signals has been investigated. Figure 2g shows the resonance frequencies with respect to temperature. The half-integer spin (*S* = 3/2) of the silicon vacancy in 4H-SiC makes it insensitive to fluctuations in strain, temperature, and electric field^35^. The resonance frequencies are almost the same under AS and Stokes excitation conditions (the mean difference is around 0.3 ± 0.3 MHz). Different from the behavior of the divacancies in SiC^17,36^ and NV centers in diamond^37,38^, both the values are constant as the temperature increases, which is similar to low temperature results^39–41^. The results show that ZFS is almost temperature independent (see Supplementary Note 2)^39–41^, which is useful for temperature-independent quantum sensing such as magnetic field and electric field sensing in broad temperature ranges^39,41^. A comparison of ODMR contrast and linewidth as a function of temperature is displayed in Fig. 2h, i, respectively. Although the contrast for both cases decreases as temperature increases, the AS excited ODMR contrast is approximately four times that of Stokes as the temperature rises from 297.2 to around 421 K. Moreover, the AS excited ODMR signal can still be detected at 500 K, while the Stokes excited ODMR signal is undetectable at the same experimental conditions. This property is useful for high-temperature quantum sensing, which is not only of interest in extending the scope of quantum sensing but also of practical applications in many important fields of nanotechnology^42^. Distinct to ODMR contrast, both linewidths are almost constant, with the AS excited ODMR linewidth also smaller than the Stokes excitation one. The results demonstrate that the AS excited ODMR signals are more robust than that of the Stokes excitation.

**Fig. 3:**
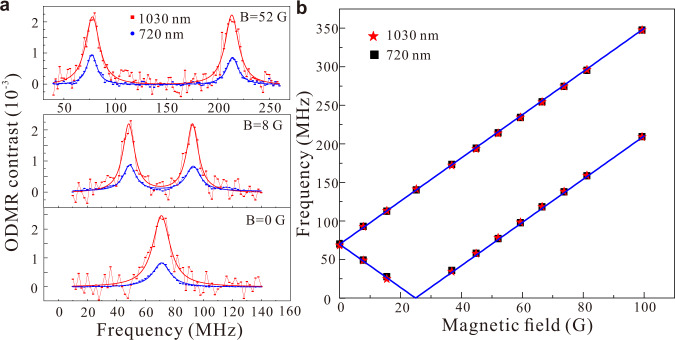
Stokes and AS excited ODMR spectra of V_Si_ defects for different external magnetic fields at room temperature. **a** Three representative Stokes (blue) and AS excited (red) ODMR spectra of V_Si_ defects for the same Rabi frequency (0.8 MHz) at different *c*-axis external magnetic fields. **b** Stokes (black) and AS excited (red) ODMR resonance frequencies as a function of the *c*-axis magnetic field.

After optimizing the AS excited ODMR spectrum, we then studied its magnetic field-dependent behavior, which is the basis of quantum sensing. Under an external magnetic field, there are two dipole-allowed transitions $|-1/2\rangle \leftrightarrow |-3/2\rangle$ and $|1/2\rangle \leftrightarrow |3/2\rangle$, with corresponding transition frequencies of $|2D-g{\mu }_{\rm{B}}B|$ and $|2D+g{\mu }_{\rm{B}}B|$, respectively. The parameters *D*, *g*, *μ*_B_, and *B* represent ZFS, Lande *g* factor, Bohr magneton, and external magnetic field, respectively. Figure 3a exhibits the Stokes and AS excited ODMR spectra for three different *c*-axis magnetic fields. For convenience, the laser powers for the Stoke and AS cases are set at 1 and 14 mW, respectively, while the MW power is set to be the same. Both Stokes and AS excited ODMR spectra show the same splitting; however, contrast under AS excitation is approximately three times that of Stokes excitation. For example, at the magnetic field of 52 G, the Stokes and AS excited ODMR resonance frequencies are 77.4 (77.8) and 214.1 (213.8) MHz, respectively. A summary of both Stokes and AS excited ODMR resonance frequencies at various magnetic fields is shown in Fig. 3b. The blue lines are the calculated transition frequencies. All the measured resonance frequencies of Stokes and AS excited ODMR coincide and agree well with the calculated results. As with Stokes excited ODMR, the demonstrated AS excited ODMR technology can also be used for direct current magnetic field sensing^15^.

**Fig. 4:**
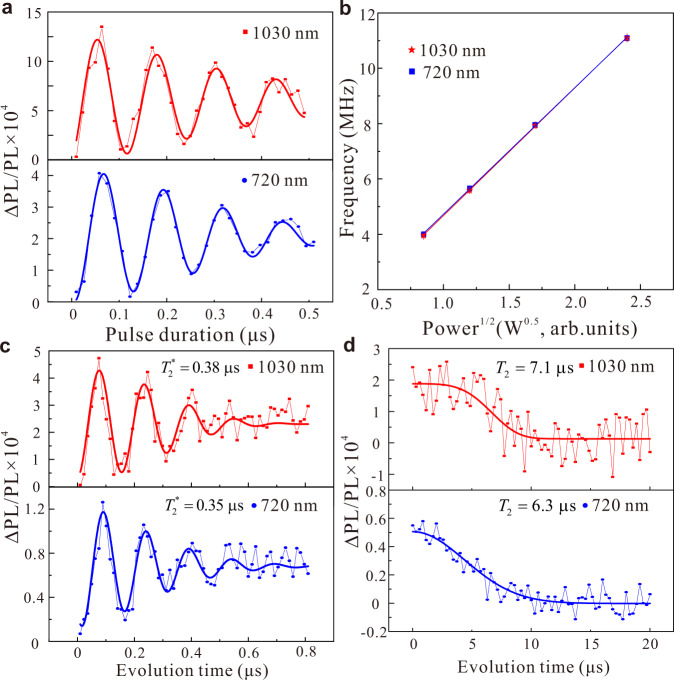
Coherent control of V_Si_ defects at room temperature under Stokes and AS excitation. **a** Measurement of Rabi oscillation under Stokes (blue) and AS (red) excitation at the same MW power at 52 G. **b** Measured Rabi frequencies as a function of MW power under Stokes (blue) and AS (red) excitation. The lines are the linear fits to the data and match with each other. **c**, **d** Comparison of the Ramsey and spin echo measurements under Stokes (blue) and AS (red) excitations, respectively.

High-quality coherence properties of solid-state spin qubits are significant for quantum computing^8–10^ and high-sensitivity quantum sensing^16,17^. In view of this, we study the coherence properties of V_Si_ defects under AS excitation. The experimental coherence control pulse sequences are the same for both the Stokes and AS excitations, which are also the same to the previous standard pulse sequences^8,17,25^ (see Supplementary Note 3). As shown in Fig. 4a, the Rabi oscillations under Stokes (blue) and AS (red) excitations are measured using a resonant frequency of 77.5 MHz at 52 G with standard Rabi pulse sequences at room temperature^17,25^. From the fit, the AS excited Rabi frequency is 8.01 ± 0.09 MHz, which is the same as that of Stokes (7.94 ± 0.05 MHz). The contrast of the AS excited Rabi oscillation is around three times that of the Stokes results at the same time. Figure 4b presents the calculated Rabi frequencies at different MW power under Stokes (blue) and AS (red) excitation, respectively. The frequencies are almost the same and linearly increase with the square root of the MW power. The results demonstrate that Rabi oscillation follows the same behavior under MW fields for both Stokes and AS excitations.

To measure the inhomogeneous spin-dephasing time under AS excitation, we employ the standard Ramsey pulse sequence. The comparison of Stokes (blue) and AS (red) excited Ramsey oscillations is presented in Fig. 4c. The spin dephasing time *T*_2_^*^ for the AS (Stokes) excitation is 0.38 ± 0.05 μs (0.35 ± 0.04 μs), respectively. The experiments show that the AS excitation method not only does not change the spin-dephasing time but also can increase the signal contrast by approximately three times. We subsequently investigate the AS excited spin coherence time using the spin echo pulse sequence. The AS excited coherence time *T*_2_ is found to be 7.1 ± 0.6 μs, which is also consistent with the Stokes value of 6.3 ± 0.4 μs. As with dephasing time, spin coherence time is not affected by the AS excitation method. The comparison of coherent control under Stokes and AS excitations demonstrates that the AS excitation technology does not affect the spin coherence properties. Moreover, it can also increase the signal contrast about three times, which is beneficial for experiments. In addition, coherent control of single V_Si_ defects may also be realized using photon structures at a higher temperature.

### Theoretical analysis on the ODMR contrast

**Fig. 5:**
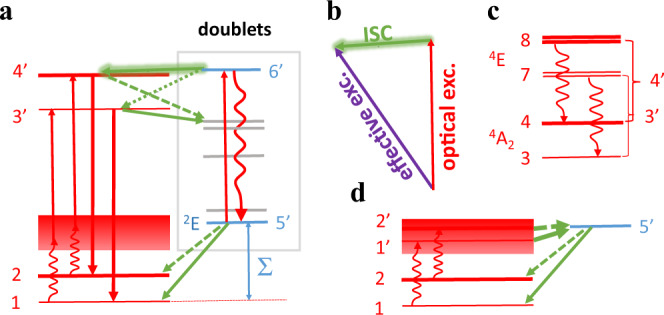
Anti-Stokes excitation scheme of V_Si_ defects. **a** Energy diagram of the quartet (red horizontal lines) and doublets. The position of the doublets (blue and gray horizontal lines) is not exactly known. The energy differences are not linearly scaled for the sake of clarity, which includes the ionic relaxations in each multiplet (ZPL energies). The shaded area is the phonon sideband of states 1 and 2. The lowest energy doublet (^2^E) is shown among the other doublets. Σ is the energy difference between the ground state manifold and the ^2^E level (state 5’). A predominant spin-selective intersystem crossing (ISC) occurs between a resonant doublet state and 4’ (*m*_s_ = ±3/2 manifolds). **b** The optical excitation (red arrow) between the doublets ^2^*E* (state 5’) and the highest doublet close to resonance with the quartet levels (state 6’) followed by a fast ISC (shaded green arrow) toward the *m*_s_ = ±3/2 states due to spin–orbit coupling leads to an effective spin-flipping optical excitation (purple arrow). There might be a weaker ISC toward the *m*_s_ = ±1/2 states (green dotted arrow) too. **c** The nature of 3’ and 4’ states is depicted, which are the phonon coupled *m*_s_ = ±3/2 (3 and 7) and *m*_s_ = ±1/2 states (4 and 8) from the ^4^A_2_ and ^4^E quartet excited states, respectively. **d** The high energy vibronic states 1’ and 2’ of states 1 and 2, respectively. ISC with enhanced rates may occur from states 1’ and 2’ to state 5’ beside the optical excitation toward states 3’ and 4’, respectively.

To make the explanation for the increased ODMR contrast for the AS approach as compared to the Stokes approach more clear, we first discuss the relationship of the continuous wave (CW) ODMR contrast between the decay rates with the energy levels shown in Figs. 1a and 5 that we took from first principles calculations^43^. We label the states with *m*_s_ manifolds by numbers (see also Supplementary Note 1). For the case of Stokes excitation, radiative decay occurs from state 3 to state 1 and from state 4 to state 2 with the rate of *r*_d_. The corresponding intersystem crossing (ISC) rates between states *i* and *j* are labeled by *r*_*ij*_. The occupation of *i* level is labeled by *n*_*i*_. The total intensity of fluorescence $I={I}_{\pm 1/2}+{I}_{\pm 3/2}$, where

1 $${I}_{\pm 1/2}=\frac{{n}_{1}{c}_{1}{r}_{{\rm{d}}}}{{r}_{{\rm{d}}}+{r}_{35}}$$

and

2 $${I}_{\pm 3/2}=\frac{{n}_{2}{c}_{1}{r}_{{\rm{d}}}}{{r}_{{\rm{d}}}+{r}_{45}}$$

where *c*_1_ is the rate of photo-excitation. In the weak illumination limit, the electron dominantly occupies the ground state spin manifolds, i.e., *n*_1_ + *n*_2_ ≈ 1, where the spin-polarization *P* between the states can be expressed as *n*_1_ − *n*_2_ ≈ *P* because *m*_s_ = ±1/2 spin state is spin-polarized according to previous measurements^44^. Upon illumination with a strength of *c*_1_ < *r*_d_, the CW ODMR contrast *C* may be expressed as

3 $$C=\frac{I(P=0)}{I(P=1)}-1$$

where Eq. (3) together with Eqs. (1) and (2) simplifies to

4 $$C=0.5\frac{{r}_{35}-{r}_{45}}{{r}_{{\rm{d}}}+{r}_{45}}$$

A small positive ODMR contrast is developed by the condition of *r*_35_ > *r*_45_, i.e., a faster ISC process from the *m*_s_ = ±1/2 manifold than from the *m*_s_ = ±3/2 manifold toward the doublet states, which is observed in the experiments.

In the AS excitation, we suppose the simple phonon relaxation and excitation processes should be spin-conserving. As shown in Fig. 1a, the probability to get from the quartet ground state manifold to the quartet excited state manifold is less than that in the Stokes excitation (labeled as *c*_1_) because it involves the phonon excitation in the former process. Once the electron decays from the quartet excited state to the lowest energy doublet state, which is a metastable state with relatively long lifetime, the CW laser used for AS excitation may excite the electron from the metastable doublet state, selectively, to state 4 (*m*_s_ = ±3/2 spin state). The process is represented by a purple arrow in Figs. 1a and 5b, which competes with the decay of the electron from the metastable doublet state to the quartet ground state by ISC. In this scenario, the brighter spin state can be increasingly populated in the quartet excited state manifold (state 4), which leads to an enhanced CW ODMR readout contrast (see also Supplementary Note 1).

The effective spin-flipping optical excitation may be explained by a relatively strong optical excitation between the doublets followed by a spin-selective ISC toward the *m*_s_ = ±3/2 spin states (see Fig. 5b). The refined model is depicted in Fig. 5a. According to first principles calculations beyond density functional theory^43^, the lowest energy doublet level (^2^E) is at about Σ = 0.15 eV above the quartet ground state’s level, which is explicitly given and labeled as state 5’. The AS excitation energy is able to promote this electron to state 6’, which is higher energy doublet state and quasi-degenerate with the quartet levels, depicted explicitly in the series of other doublets. We assume that there is a dominant relaxation path from state 6’ toward the *m*_s_ = ±3/2 spin states, which is further considered in our analysis. The exact nature of state 6’ is not known at the moment from literature^43^ but may be associated with a doublet ^2^E counterpart of the ^4^E quartet (see the discussion in Supplementary Note 1). The ^4^E state is the second optically allowed excited state that is separated from the ^4^A_2_ state by about 20 meV, and they are strongly coupled by phonons^45^ (see Fig. 5c). We label the corresponding spin states of ^4^E manifold as 7 and 8. As the phonon coupling is strong and the phonon decay process is considered to be rapid between states 8 and 4 as well as states 7 and 3 (c.f., Figs. 1a and 5c), the electron then decays either from state 4 or state 3 toward the lower energy doublets by ISC or toward the ground state manifold by emitting photons. Therefore, the corresponding joint states are labeled as 4’ (*m*_s_ = ±3/2 states) and 3’ (*m*_s_ = ±1/2 states) in Fig. 5a.

The CW ODMR contrast changes because the optically excited bright *m*_s_ = ±3/2 spin state can be occupied from the doublets in AS excitation as a new source of the occupation of that state with respect to that of the Stokes excitation. We show this effect briefly by calculating the CW ODMR contrast upon AS illumination with the model sketched in Fig. 5. For the sake of simplicity, the prime labels are omitted in the following equations that describe the possible decay and excitation routes. *I*_±1/2_ in Eq. (1) does not change. However, *I*_±3/2_ modifies to

5 $${I}_{\pm 3/2}=\frac{{n}_{2}{c}_{1}{r}_{{\rm{d}}}+{n}_{6}{r}_{64}{r}_{{\rm{d}}}}{{r}_{{\rm{d}}}+{r}_{45}}$$

where *r*_64_ is the ISC rate from state 6’ to state 4’. The occupation of level 6’ (*n*_6_) depends on the occupation of level 5’ (*n*_5_) and the strength of optical excitation toward state 6’ (*c*_56_), which should be much higher than *c*_1_ in the AS excitation because it does not require the absorption of phonons (see also Supplementary Note 1). The occupation of levels 5’ and 6’ in the steady state may be given as

6 $${n}_{5}=\frac{{n}_{3}{r}_{35}+{n}_{4}{r}_{45}}{{r}_{51}+{r}_{52}+{c}_{56}}$$

and

7 $${n}_{6}=\frac{{n}_{5}{c}_{56}}{{r}_{63}+{r}_{65}}$$

By assuming that *r*_64_ is large (fast ISC because of the resonance conditions and sufficiently large spin–orbit interaction)^45^, one can again assume *n*_1_ + *n*_2_ ≈ 1 and *n*_1_ − *n*_2_ ≈ *P* similarly to the case of Stokes excitation. By combining Eqs. (1), (6), and (7), one can express Eq. (5) as a function of *n*_1_ and *n*_2_, where the contrast is again defined by Eq. (3). Finally, the calculated CW ODMR contrast of AS excitation will be larger than that for Stokes excitation because *I*(*P* = 1/2) is unchanged but *I*(*P* = 3/2) increases because of the increase in *I*_±3/2_. We note that this basic result is not altered by considering a weak scattering from the state 6’ toward 3’ (*r*_63_ < *r*_64_), which is ignored now for the sake of clarity.

We further note that an additional effect may contribute to the enhanced CW ODMR contrast upon AS excitation (see Fig. 5d). Because the system is spin-polarized to state 1, it is plausible to assume that *r*_51_ > *r*_52_, i.e., continuous green arrow vs. dotted green arrow in Fig. 5a. In the AS excitation of the ground state manifold, a photon is absorbed first, thus we arrive at state 1’ from state 1 and at state 2’ from state 2. If the intensity of the pumping laser is not sufficiently high, then ISC from states 1’ and 2’ to state 5’ may conquer with the optical excitation from states 1’ and 2’ to states 3’ and 4’, respectively, where ${r}_{51{\prime} } > {r}_{52{\prime} }$ because of the assumption of *r*_51_ > *r*_52_ and the spin–orbit coupling—responsible for ISC between the doublet and the quartet—is not affected directly by phonons (see Supplementary Note 1 for details). As a consequence, the occupation of state 3’ is effectively depleted with respect to that of state 4’, and thus *I*_±1/2_ effectively decreases over *I*_±3/2_ with respect to the case of Stokes excitation. This effect may increase the CW ODMR contrast of AS excitation compared to that of Stokes excitation.

## Discussion

In conclusion, we realized the coherent control of V_Si_ defect spin ensembles in 4H-SiC under AS excitation at room temperature. Through investigation of the AS PL dependence on laser power and temperature, we demonstrate that the AS PL is induced by the phonon-assisted single low-energy photon absorption process. Moreover, the results show that the AS PL can be used for all-optical high-temperature temperature sensing. Based on stable AS PL, a comparison of Stokes and AS excited ODMR signals as a function of laser power, MW power, and temperature was demonstrated, which both show similar behaviors. In addition, the AS excited ODMR contrast is several times larger than the Stokes excitation contrast, while the AS excited ODMR linewidth is smaller than the Stokes excitation one. The former might be explained by a resonant spin-flipping optical transition between the doublet metastable and excited state quartet, which implies the position of the doublet metastable level with respect to the ground state quartet at around 0.15 eV, in accordance with a recent ab initio study^43^. Furthermore, the AS excited ODMR signals are also more robust than Stokes excitation one at high temperatures, making it more suitable for quantum technologies at elevated temperatures. The measurement of Stokes and AS excited ODMR spectra at various magnetic fields shows that they have the same resonant frequency. On this basis, we realized the coherent control of defect spin under AS excitation at room temperature. The results demonstrate that the AS excitation methods not only preserve spin properties but also can improve the signal contrast around three times. Moreover, the AS excitation methods can also directly be used for other solid state qubits, including NV centers in diamond^20^ and divacancies^8^ and NV centers^25,26^ in SiC, etc. The experiments form a framework for developing AS excitation technologies in quantum information processing and quantum sensing.

## Methods

### Sample preparation

In the experiment, we use a high-purity 4H-SiC epitaxy sample. Due to the low AS exciting efficiency, high-density V_Si_ defect ensembles are required to obtain detectable signals. In view of this, we implanted 20 keV helium ions (dose 1 × 10^13^/cm^2^) and subsequently annealed the sample at 500 °C for 2 h.

### Experimental set-up

In the experiment, a home-made confocal system combined with a MW system is applied to excite and manipulate V_Si_ defects. Two lasers with wavelengths of 720 and 1030 nm, which are modulated by two separate acousto-optic modulators, are used to excite and polarize V_Si_ defects, respectively. In order to efficiently collect V_Si_ defect fluorescence, a non-polarizing beam splitter with 30/70 beam splitting ratio (Thorlabs, BS080) is used to separate the excitation and collection paths. For the room temperature ODMR and coherent spin control experiments, a 1.3 N.A. oil objective is used to efficiently collect the fluorescence. For the AS excited PL at low and high temperatures, a 0.65 N.A. air objective is used. In order to collect the V_Si_ defect fluorescence under both Stokes and AS excitation, we simultaneously used an 850-nm long-pass filter and a 1000-nm short-pass filter. The low-temperature experiment is performed using a Montana cryostation (4–350 K) combined with a confocal system. For the high-temperature experiments, the sample temperature is controlled and readout by a metal-ceramic heater (HT24S, Thorlabs) and a resistive temperature detector (TH100PT, Thorlabs), respectively. In the ODMR experiments, the fluorescence is collected by a multimode fiber into a femtowatt photoreceiver (OE-200-Si). For the readout of the ODMR signal, standard lock-in methods are used in the experiment, which is the same to the previous experiments^8,17,25,27^. The ODMR contrast is defined as ΔPL/PL = *V*_Mod_/*V*_Tot_, where *V*_Mod_ is the magnitude of the 70 Hz modulated component of the output voltage and *V*_Tot_ is the total time-averaged output voltage, which is the same with previous lock-in methods^8^.

## Supplementary information

Supplementary Information

## Data Availability

The data that support the findings of this study are available from the corresponding author upon reasonable request.
