# Supplementary material for: Robust coherent control of solid-state spin qubits using anti-Stokes excitation
Source: Nat Commun. 2021 May 28;12:3223. doi: 10.1038/s41467-021-23471-8 (PMC8163787; doi:10.1038/s41467-021-23471-8)
Supplement: Supplementary file 1 — Supplementary Information [file 41467_2021_23471_MOESM1_ESM.pdf]

Supplementary information for:

Robust coherent control of solid-state spin qubits using anti-Stokes excitation

Jun-Feng Wang[1, 2], Fei-Fei Yan[1, 2], Qiang Li[1, 2], Zheng-Hao Liu[1, 2], Jin-Ming Cui[1, 2], Zhao-Di Liu[1, 2,], Adam Gali[3,4*], Jin-Shi Xu[1, 2,*], Chuan-Feng Li[1, 2,*] and Guang-Can Guo[1, 2]

[1]CAS Key Laboratory of Quantum Information, University of Science and Technology of China, Hefei, Anhui 230026, People's Republic of China

[2]CAS Center for Excellence in Quantum Information and Quantum Physics, University of Science and Technology of China, Hefei, Anhui 230026, People's Republic of China

[3]Department of Atomic Physics, Budapest University of Technology and Economics, Budafokiút. 8, H-1111 Budapest, Hungary.

[4]Wigner Research Centre for Physics, P.O. Box 49, H-1525 Budapest, Hungary.

[*]Corresponding author: gali.adam@wigner.hu, jsxu@ustc.edu.cn, cfli@ustc.edu.cn

Supplementary Note 1 ODMR detection under Stokes excitation

The ODMR contrast depends on the intrinsic properties of the defect and external parameters too. Such external parameters consist of technicalities (such as the optical excitation power, microwave power, etc.) and materials issues (background luminescence due to other defects, defect-defect interaction via ionisation and other processes). The intrinsic properties of the defect qubit include the basic electronic structure, electron-phonon interaction and spin-orbit interaction that are responsible for the radiative decay and non-radiative decay from the optical excited states[1]. In our analysis in the main text, we focus on the intrinsic properties of the V_{Si} in 4H-SiC. We assume that the continuous-wave (CW) laser excitation leads to relatively weak illumination per defect meaning that the ground state spins manifold is dominantly occupied for the ensembles of V_{Si} defects in the process.

The analysis starts with the basic electronic structure of the defect. The defect introduces three levels in the gap with a_1 symmetry and {a_1, e} symmetries where the latter is a pseudo triply degenerate state (t_2 in the cubic polytype of SiC) which splits to a_1 and e levels due to the C_{3v} symmetry of the 4H SiC crystal field[2]. In order to distinguish the two a_1 states, those are often labelled as u and v in the literature beside the double degenerate e level. In the negative charge state, the defect levels are occupied by five electrons. The ground state electronic configuration is $u(2)v(1)e(2)$, i.e., the lowest a_1 state is fully occupied whereas the other three electrons may reside parallel to each other which establishes S=3/2 electron spin state. This manifold can be shortly described as $v(1)e(2)$ by explicitly writing only the open shell defect levels. By promoting an electron from u to v level, i.e., $u(1)v(2)e(2)$ electron configuration or shortly $u(1)e(2)$, a higher energy S=3/2 state can be formed. Another excited state S=3/2 can be achieved by $u(1)v(1)e(3)$ electron configuration or $u(1)v(1)e(1)$ configuration in the hole picture.

Indeed, the defect has S=3/2 ground and optically accessible excited states with m_s=±1/2 and m_s=±3/2 spin manifolds. The ground state and the lowest energy S=3/2 excited state have $^4A_2$ symmetry whereas the higher energy quartet has ^4E symmetry[3].

The usual excitation scheme when the excitation energy is larger than the zero

phonon line (ZPL) energy, the so-called Stokes excitation (see Fig. 1**a** in the main text). In this case, one may use a five-level scheme where the series of doublet states is substituted by a single effective doublet state (state 5) where the electron may scatter from the excited state spin manifolds (states 3 and 4, i.e., m_s=±1/2 and m_s=±3/2, respectively) to this state or can scatter from this state to the ground state spin manifolds (states 1 and 2, i.e., m_s=±1/2 and m_s=±3/2, respectively) via intersystem crossing (ISC). The ISC is responsible for the observed optically detected magnetic resonance (ODMR) readout contrast and the optical spin-polarisation in the quartet ground state. As we argued in the main text, the doublet states play a crucial role also in the anti-Stokes excitation of the defect. Unfortunately, the nature of the doublet states is still largely unexplored. Although, a detailed group theory analysis about the quartet and dark doublet states and the possible intersystem crossing routes have been recently reported but the positions and the order of doublet levels have not yet been explicitly determined [3]. In a recent study, the vertical excitation energies of the quartet and doublet levels between the quartet levels in V_{Si} in 4H-SiC have been reported in a theoretical study but the doublet levels above the excited states' quartet levels were not listed[4], and the final position of the electronic levels may shift by taking the ionic relaxations in each multiplet into account. Furthermore, electron-phonon coupling may also mix these electronic doublet states similarly to the $^4A_2$ and ^4E excited states[5] which can seriously affect the corresponding ISC rates. In the lack of direct *ab initio* data, one has to rely on group theory considerations[3] and interpret the experimental results based on the selection rules.

In the followings, we use the labels and definitions from Ref. 3 in the discussion. The ISC goes with $\lambda F(E)$ where λ is the spin-orbit coupling between the quartet and doublet state, E is the energy difference between these levels and F is the phonon overlap spectral function at this energy. Depending on the symmetry of the quartet and doublet states, the parallel $\lambda_{||}$ (m_s quantum is preserved) or the perpendicular $\lambda_{\perp}$ component (m_s quantum number shifts) of the spin-orbit coupling may be allowed. We add our comment here to the results in Ref. 3 that the *u* orbital is very much an atomic *s*-like function whereas the *v* orbital is rather a p_z-like function, thus the spin-orbit coupling

between u and e orbitals ($p_{\{x,y\}}$-like function), labelled as $\lambda_{\perp,2}$, should be weak compared to that of v and e orbitals, labelled as $\lambda_{\perp,1}$, in Ref.3. Another consideration is that the ionic relaxations are relatively small between such states that share the same electron configurations. In this case, $F(E)$ is larger for smaller E, i.e., energy spacing between the initial and final states in ISC. As a consequence, the ISC rate is higher between 1'/2' and 5' states than that between 1/2 and 5 states (see Fig 5d in the main text).

In the proposed anti-Stokes excitation scheme, an electron can be excited from the metastable doublet state 5' to an excited doublet state 6' from which a dominant spin-selective ISC should occur towards the bright m_s=±3/2 manifold of the excited quartet states (see Fig. 5a of the main text). According to Ref. 3, the ^{2}E doublet with $u(1)v(1)e(1)$ character (labelled as d6 function there) is connected to the m_s=±3/2 $^4A_2$ manifold by $\lambda_{\perp,1}$. Indeed, it is likely that the ^{2}E doublet with $u(1)v(1)e(1)$ character should reside close in energy to ^{4}E level which has also an $u(1)v(1)e(1)$ character and it lies above the $^4A_2$ level by ~20 meV, so the energy spacing between $^4A_2$ level and this ^{2}E level should be small which makes $F(E)$ in ISC rate sizeable. On the other hand, $\lambda_{||}$ connects this ^{2}E state to m_s=±1/2 ^{4}E manifold too which goes against the occupation of m_s=±3/2 state. Thus, this ^{2}E level should either reside between the $^4A_2$ and ^{4}E levels to be effective only towards m_s=±3/2 $^4A_2$ state or it lies above ^{4}E level but then $\lambda_{\perp,1}$ should be greater than $\lambda_{||}$ for dominant occupation of m_s=±3/2 $^4A_2$ state. Both scenarios are principally feasible which may explain the enhanced ODMR contrast upon anti-Stokes excitation.

Supplementary Note 2 Additional experimental data

We also measure the PL confocal images of V_{Si} defects in 4H-SiC under AS excitation. Four 10 × 10 μm^2 confocal images under AS excitation at different low temperatures are displayed in Fig. S1. We can see that the AS excited counts slowly increase as the temperature increases from 11 to 223 K, and then dramatically increase when the temperature increases to 297 K.

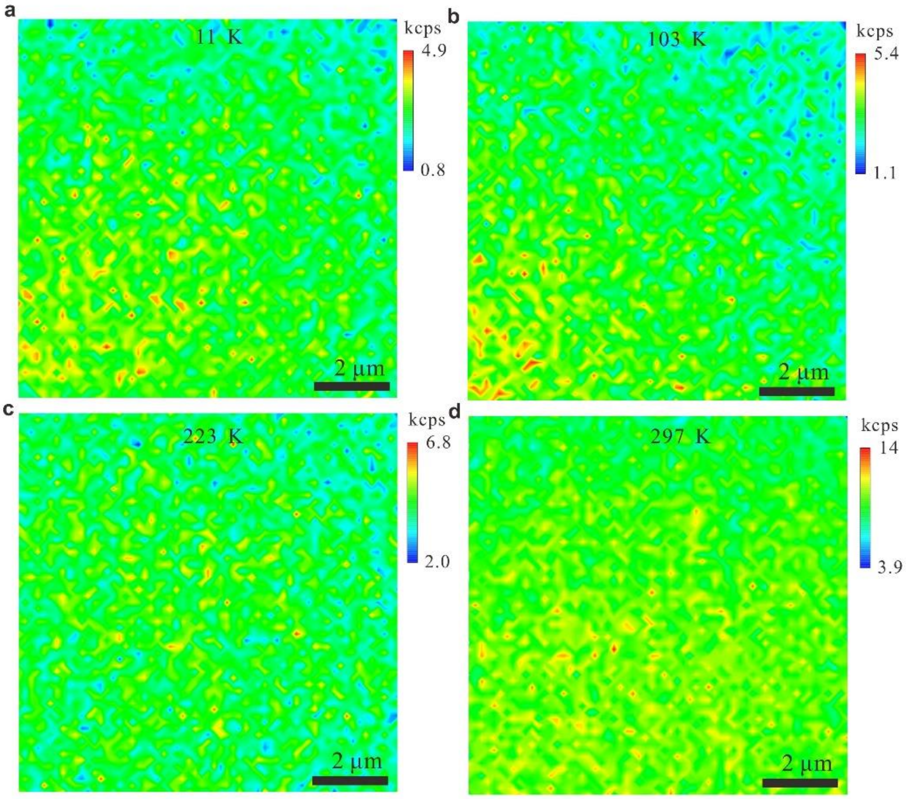

Supplementary Figure 1. **AS excited PL confocal scans. a-d,** AS excited PL confocal images (10 × 10 μm^2) of V_{Si} defects at four different temperatures.

In Fig. S2a, we present the AS excited counts as a function of laser power at different low temperatures. All counts linearly increase as laser power increases, which demonstrate the AS PL is due to the phonon-assisted single photon emission process. Moreover, we can see that the slope increases slowly as the temperature increases from 11 to 210 K, and increases quickly as the temperature increases to 319 K. In addition to count tracing at maximum laser power for different temperatures in the main text, we also studied tracing for different laser powers at room temperature. As shown in Fig. S2b, the counts are stable for three different laser powers, which demonstrates the robustness of the AS excitation of V_{Si} defects. We then measured the Stokes excited counts as a function of laser power at four different high temperatures (Fig. S2c). The saturation counts decrease as temperature increases.

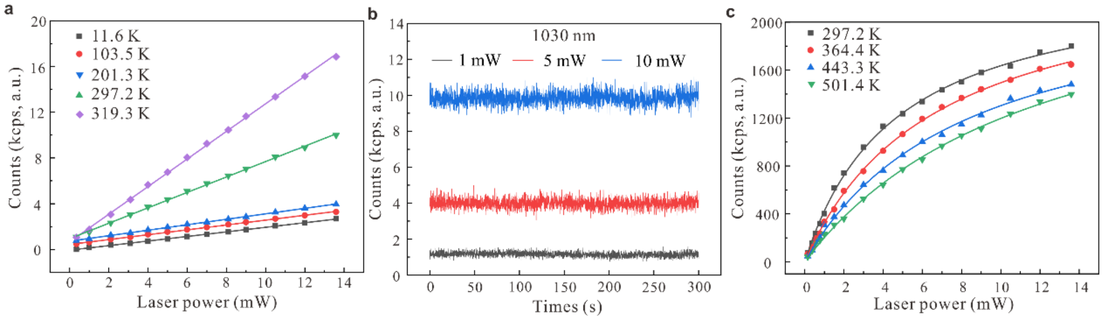

Supplementary Figure 2. **AS excited counts at different temperature. a,** AS excited counts as a function of the laser power at different low temperatures. **b,** Time trace of AS excited counts for different laser powers at room temperature. **c,** The Stokes excited counts as a function of laser power at different high temperatures.

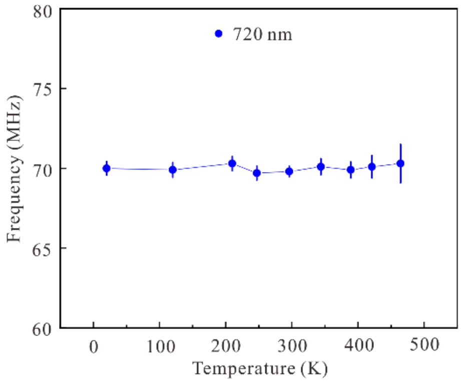

Supplementary Figure 3. **ZFS measurements.** ZFS of V_{Si} defects under Stokes excitation as the temperature increases from 11 K to 500 K. Error bars represent the standard deviations of the corresponding fittings.

Finally, as presented in Fig. S3, we investigate the zero field splitting (ZFS) of V_{Si} defects as a function of temperature under the Stokes excitation. ZFS is almost constant as the temperature increases from low (11 K) to elevated temperature (501 K), which is the same with previous results and silicon vacancy in 6H-SiC[6-8]. The temperature independent of ZFS is important for temperature-independent DC magnetic field sensing.

Supplementary Note 3 The pulse sequences for coherent control

The pulse sequence is same for both the Stokes and anti-Stokes excitation. As shown in Fig. S4, for the Rabi and Ramsey experiment the laser pulse for both polarization and readout is 5 μs, while for the spin echo, the laser pulse length for polarization and readout is 10 μs. Both the pulse lengths are the same as previous pulse lengths[9-11].

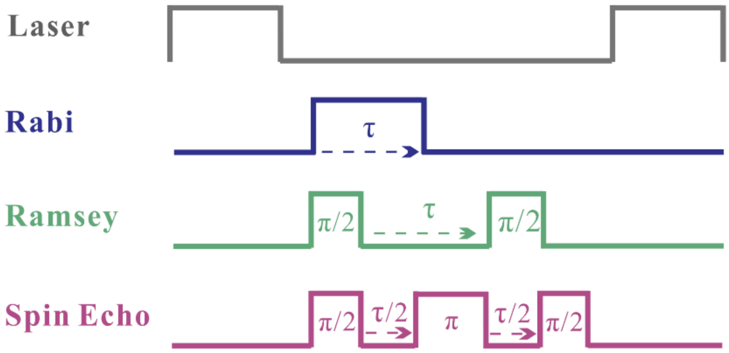

Supplementary Figure 4. **Experiments pulse sequences.** Laser and microwave pulse sequences of the Rabi, Ramsey oscillations and the spin echo in the main text.

References

1. Gali, A., Ab initio theory of the nitrogen-vacancy center in diamond. Nanophotonics 8, 1907–1943 (2019).

2. Erik Janzén, et al. The silicon vacancy in SiC. Physica B 404, 4354–4358 (2009).

3. Dong, W. Z., Doherty, M. W., Economou. S. E. Spin polarization through intersystem crossing in the silicon vacancy of silicon carbide. Phys. Rev. B 99, 184102 (2019).

4. Widmann, M., et al., Electrical charge state manipulation of single silicon vacancies in a silicon carbide quantum optoelectronic device. Nano Lett. 19, 7173–7180 (2019).

5. Udvarhelyi, P., et al. Vibronic states and their effect on the temperature and strain dependence of silicon-vacancy qubits in 4H-SiC. Phys. Rev. Appl. 13, 054017 (2020).

6. Anisimov, A. N., et al. Optical thermometry based on level anticrossing in silicon carbide. Sci. Rep. 6, 33301 (2016).

7. Shang Z., et al. Local vibrational modes of Si vacancy spin qubits in SiC. Phys. Rev. B 101, 144109 (2020).

8. Kraus, H., et al. Magnetic field and temperature sensing with atomic-scale spin

defects in silicon carbide. Sci. Rep. 4, 5303 (2014).
9. Koehl, W. F., et al. Room temperature coherent control of defect spin qubits in silicon carbide. Nature 479, 84–87 (2011).
10. Zhou, Y., et al. Self-protected thermometry with infrared photons and defect spins in silicon carbide. Phys. Rev. Applied 8, 044015 (2017).
11. Wang, J. F., at al. Coherent control of nitrogen-vacancy center spins in silicon carbide at room temperature. Phys. Rev. Lett. 124, 223601 (2020).
